# Evaluation of gastrointestinal bacterial population for the production of holocellulose enzymes for biomass deconstruction

**DOI:** 10.1371/journal.pone.0186355

**Published:** 2017-10-12

**Authors:** Dhaneshwaree Asem, Vincent Vineeth Leo, Ajit Kumar Passari, Mary Vanlalhruaii Tonsing, J. Beslin Joshi, Sivakumar Uthandi, Abeer Hashem, Elsayed Fathi Abd_Allah, Bhim Pratap Singh

**Affiliations:** 1 Molecular Microbiology and Systematics Laboratory, Department of Biotechnology, Aizawl, Mizoram University, Mizoram, India; 2 Bioctalysts Lab., Department of Agricultural Microbiology, Tamil Nadu Agricultural University, Coimbatore, India; 3 Botany and Microbiology Department, College of Science, King Saud University, Riyadh, Saudi Arabia; 4 Plant Production Department, College of Food and Agricultural Sciences, King Saud University, Riyadh, Saudi Arabia; Universite Paris-Sud, FRANCE

## Abstract

The gastrointestinal (GI) habitat of ruminant and non-ruminant animals sustains a vast ensemble of microbes that are capable of utilizing lignocellulosic plant biomass. In this study, an indigenous swine (Zovawk) and a domesticated goat (Black Bengal) were investigated to isolate bacteria having plant biomass degrading enzymes. After screening and enzymatic quantification of eighty-one obtained bacterial isolates, *Serratia rubidaea* strain DBT4 and *Aneurinibacillus aneurinilyticus* strain DBT87 were revealed as the most potent strains, showing both cellulase and xylanase production. A biomass utilization study showed that submerged fermentation (SmF) of D2 (alkaline pretreated pulpy biomass) using strain DBT4 resulted in the most efficient biomass deconstruction with maximum xylanase (11.98 U/mL) and FPase (0.5 U/mL) activities (55°C, pH 8). The present study demonstrated that bacterial strains residing in the gastrointestinal region of non-ruminant swine are a promising source for lignocellulose degrading microorganisms that could be used for biomass conversion.

## Introduction

Lignocellulose biomass (LCB), such as agricultural and forest residues, are considered to be a potential feedstock for the generation of renewable bioenergy [1, 2]. Bioenergy has gained immense interest, primarily because of concerns such as the depletion of non-renewable fossil fuels, global warming and the universal call for cleaner environments [3–5]. LCB can serve as an economical source of fermentable sugar for second generation bioethanol production [6, 7]. In addition, LCB represents the most abundant organic matter in nature, composed of an interlinked matrix of cellulose and hemicellulose along with glycosylated proteins and lignin polymer moieties [8] that resist bioconversions. Various strategies have been developed to improve the biomass conversion efficiency. However, significant challenges still exist for reducing cost of enzymes, optimizing biorefinery processes and developing simultaneous saccharification. The study of natural biocatalyst systems will help us to address these challenges, as many natural biocatalyst systems have evolved to convert lignocellulosic biomass with high efficiency [9–11]. Microorganisms isolated from nature and the guts of herbivorous animals can degrade different feedstocks. Larger animals lack the capacity to degrade lignocellulosic materials themselves and instead rely on their gut microbial communities [12].

Cellulases produced by microorganisms have been divided into three major classes: cellobiohydrolase (EC 3.2.1.91); endo-glucanase (EC 3.2.1.4) and β-glucosidase (EC 3.2.1.21) [13]. However, the complete hydrolysis of xylan requires an enzyme system consisting of endo-1,4-*β*-xylanases (EC 3.2.1.8), *β*-_D_-xylosidases (EC 3.2.1.37), *α*-L-arabinofuranosidases (EC 3.2.1.55), *α*-glucuronidases (EC 3.2.1.139), acetyl xylan esterases (EC 3.1.1.72), and ferulic/coumaric acid esterases (EC 3.1.1.73) [14, 15]. Although fungal strains have been commercially used for the production of cellulase, bacterial cellulases have several advantages over these enzymes. For example, bacteria have a faster growth rate and can therefore be easily grown to high cell densities. In addition, enzyme expression systems in bacteria are more convenient and bacteria can withstand extreme conditions, such as high or low temperatures and alkaline or acidic environments [16]. These enzymes are well-studied for their several biotechnological applications, and in addition to their use in the bioconversion of agricultural wastes, they are used in the textile and paper industries and as additives in laundry detergents [17].

The enzymes obtained from rumen bacteria are considered potential candidates for the breakdown of cellulose [18]. Several cellulolytic and xylanolytic bacterial genera have been reported to be isolated from compost [19], swine waste [20] and hot springs [21]. In Mizoram, swine are fed with a special cellulose rich diet (chayote, *Sechium edule*), considered to be the best diet for the growth and development of the swine. However, the isolation of a bacterial population from gut of indigenous goats and swine having cellulolytic and xylanolytic potential has not yet been reported. Thus, this study aimed to isolate bacterial strains from the gut of the indigenous goat and swine having cellulolytic and hemicellulolytic potential. The strain *Serratia rubidaea* was obtained from gut of swine and showed interesting activities for the production of cellulase and xylanase, and the strain showed significant degradation of lignocellulosic biomass. The enzyme production of the bacterial strains from a ruminant and a non-ruminant organism was compared. We propose that the strain *Serratia rubidaea*, an efficient producer of both cellulase and xylanase that can be used in the degradation of biomass, could be useful for the generation of simple sugars for industrial applications.

## Materials and methods

### Sample collection

Gastrointestinal (GI) fluid was collected from an 8-month-old ruminant domesticated goat and a 7-month-old non-ruminant Zovawk (an indigenous pig of Mizoram) from a slaughterhouse in Vaivakawn, Mizoram, India (23° 43' 27" N and 92° 43' 2" E). Both animals were fed chayote (*Sechium edule*) for a month as previously described and recommended [22]. Representative samples of total GI contents (500 mL) were collected immediately after slaughter. The GI extract was collected directly from slaughterhouse and we did not deal with the animals in laboratory, the case was discussed formally in animal ethical committee and the same was approved as permit no. MZUAEC/2016-17/SLS-Biotech-26. The collected GI tract was rinsed with water and dissected using sterile scissors, and the content was transferred to a sterilized vessel. The GI fluids and solids were separated by using two layers of muslin cloth [23]. The total fluids were placed in a sterile vessel and brought to the laboratory in an icebox and processed immediately for the isolation for bacteria.

### Enumeration of the total culturable bacterial population

The total culturable bacterial populations present in the GI tracts were assessed using serial dilution and spread plate techniques. Briefly, 1 mL of GI fluid was diluted with 90 mL of double distilled water and mixed in an oscillation water bath at room temperature for 30 min. This was followed by a gradient dilution (10^−2^ to 10^−6^), and the samples were spread onto four nutritional media plates, i.e., Tryptone Soy Agar (TSA); Actinomycete Isolation Agar (AIA); Starch Casein Agar (SCA) and modified BPS-CX agar media (additional 1% lignin). All media were supplemented with 80 μg/mL of filter sterilized actidione (cycloheximide) to inhibit the growth of fungi. Plates were incubated at 37°C for 24 hrs for eubacteria and 10 to 15 days for actinobacteria. Morphologically distinct colonies were cultivated on respective media by repeated streaking and were stored on slants at 4°C. The purified cultures were also preserved for long term storage in 20% glycerol at -80°C.

### Identification and phylogenetic affiliation of isolates

The obtained isolates were grown overnight in LB broth at 37°C and genomic DNA was extracted using a Pure-link Bacterial DNA Purification Kit (Invitrogen, USA). Identification of isolates was done by amplification of 16S rRNA gene using bacteria-specific universal primers [forward primer PA (5'-AGA GTT TGA TCC TGG CTC AG-3') and reverse primer PH (5'-AAG GAG GTG ATC CAG CCG CA-3')] [24]. The PCR reaction was carried out using a Veriti thermal cycler (Applied Biosystems, Singapore). Each reaction cocktail (25 μL) contained 50 ng of genomic DNA, 10 pmol of each primer, 2.5 mM of deoxynucleotide triphosphates (dNTPs), 1X reaction buffer and 1 U/ μL of Taq DNA polymerase (Invitrogen, USA). The PCR conditions were set as follows: an initial denaturation at 94°C for 5 min, followed by 30 cycles of denaturation at 94°C for 1 min, annealing at 57°C for 1 min and an extension at 72°C for 1.2 min with a final extension at 72°C for 10 min. The amplified PCR products were visualized with a gel documentation system (Bio-rad Gel DocXR+, USA) and were purified using a Purelink PCR Purification Kit (Invitrogen, USA) and commercially sequenced at Sci-Genom Pvt. Ltd., Cochin, India.

The obtained sequences were compared with those in the GenBank and EMBL databases using BlastN and BlastX search programs, and the sequences were aligned using EMBL ProtPro [25]. The evolutionary models were selected based on lowest BIC (“Bayesian Information Criterion”) and highest AIC (“Akaike Information Criterion”) values using MEGA version 6.0. An analysis of the 16S rRNA gene sequences used the model K2+I (R = 1.24) for the construction of a neighbor-joining tree. The transition and transversion ratio (R) was calculated using MEGA version 6.0. Phylogenetic trees were constructed based on the neighbor-joining method using the Kimura 2 parameter models with MEGA 5.05 [26, 27]. The robustness of the phylogenetic trees was evaluated by bootstrap analysis with 1000 resamplings using a p-distance model [28]. Trees were viewed and edited using the program FigTree 1.3.1 (2012).

### Screening for cellulolytic and xylanolytic activity of bacteria isolates

The bacterial isolates were screened for their cellulase and xylanase production using a Congo red assay [29]. Briefly, all isolates were grown on agar plates supplemented with 0.5% (w/v) CMC and 25% (v/v) oat spelt xylan for screening of cellulases and xylanases, respectively. The plates were incubated at 30°C for 3–5 days [1], and the plates were flooded with Congo red (0.5%) for 5 min followed by destaining using 1 M NaCl for 15 min. The plates were observed for zones of clearance, where the diameter of the clear zone (halos) formed around the isolates is indicative of the magnitude of the cellulase and xylanase production [29]. The hydrolysis capacity was calculated as the ratio of the diameter of the zone of clearance divided by the diameter of the colony [30]. All cultures showing a hydrolysis capacity of ≤ 1 cm were selected for further quantitative estimates of xylanase and cellulase production.

### Quantification and optimization of enzyme production conditions

The isolates showing significant hydrolytic zones in the plate based assay were selected for quantification of cellulase and xylanase production (Leo et al., 2016). Furthermore, the effects of the incubation time (0, 12, 24, 48, 72, 96, 120 and 144 h), pH (5, 5.5, 6, 7, 8 and 9) and temperature (25, 30, 37, 45, 50, 55 and 60°C) were optimized to maximize xylanase and cellulase production.

### Cellulase and xylanase enzyme activity assays

Based on enzyme optimization and quantification results, the isolates that showed maximal cellulase and xylanase production were further characterized via FPase, CMCase and xylanase assays. For the cellulase quantification assays, the filter paper activity (FPA) and CMCase assays were performed according to Ghose [31] with certain amendments from Camassola and Dillon [32]. The FPA and CMCase activities were defined as the amount of enzyme capable of releasing 1 μmol of reducing sugar per min per mL. The xylanase activity was assessed using a previously described assay [33], with 1% xylan (w/v) supplemented as substrate. A unit of xylanase activity was defined as the amount of enzyme capable of releasing 1 μmol of xylose per min per mL. All enzyme activities were represented as U/mL. One unit (1 U) of enzyme activity was defined as the amount of enzyme that released 1 μmol of reducing sugar in one minute. All samples were analyzed in triplicate and the mean values were recorded.

### Bioconversion

Based on cellulase and xylanase enzyme assays, the best two strains were grown in 100 mL of BPS-CX broth at 30°C, 140 rpm for 3–4 days. For bioconversion studies, an abundantly available and common perennial grass, *Thysanolaena latifolia* (BPS-G104) was selected and processed [1]. The raw biomass (BPS-G104) was ground to a particle size of 0.5 to 5.00 mm and made into slurry with 20:1 ratio (biomass: sterile water). The biomass was further treated with 1% NaOH for 12 hrs at 55°C to obtain a pulpy crude biomass (BPS-G104a) [1, 34]. Subsequently, the remaining supernatant, which potentially possessed hydrolysis inhibitors, was removed using muslin cloth, then the biomass was homogenized in BPS-YM media (1% peptone and 0.5% yeast extract in phosphate buffer pH 7). The lignocellulosic components were measured for the raw and the treated samples according to the methodology of the National Renewable Energy Laboratory (NREL-TP-510-42618) to verify the effectiveness of the alkaline treatment. Submerged fermentation (SmF) was carried out using *T*. *latifolia* pulp (BPS-G104a) as the primary carbon substrate for strain DBT87 (BPS-M2). Similar treatments were performed for strain DBT4 (BPS-D2). The SmF media contained 500 mL of BPS-YM media and 20% biomass for BPS-M2 and BPS-D2. SmF was carried out for a period of 6 days at the optimized temperature (55°C) and pH (pH 8) with continuous agitation (150 rpm), and aliquots were taken on alternate days for enzyme assays. Sterile nylon cloth was used for filtering out the supernatant from the biomass pulp, which was later dried at 55°C for 48 h, and the supernatants (BPS-E4 and BPS-E87) were used as crude enzyme solutions, which were obtained from the BPS-D2 and BPS-M2 treatments, respectively. The relative dry weight (RDW) was calculated, with the alkali pretreated sample (BPS-G104a) serving as the control [1, 35].

### Fourier Transformed Infrared Spectroscopy (FTIR)

FTIR spectra of pretreated and treated biomass (BPS-M2 and BPS-D2) were obtained at room temperature, using a JASCOFT/IR-6800 spectrometer (Jasco, Japan) equipped with an attenuated total reflectance unit. Spectral data between 650 and 4000 cm^−1^ were collected, averaging 64 scans at a resolution of 4 cm^−1^. Know It All ID Expert (Biorad, USA) was used for principal component analysis (PCA).

### Observation of biomass morphology after decomposition using scanning electron microscopy (SEM)

The cell surface morphology of the pretreated and treated biomass (BPS-M2 and BPS-D2) was observed by scanning electron microscopy (SEM) as described previously [1].

### Kinetics study

The cellulase and xylanase activities of the most effective crude extracts from the biomass utilization study (DBT4) were evaluated using varying substrate concentrations of filter paper (0.1–3%) and birchwood xylan (10–100 mg) for FPase and xylanase assays. The K_m_ and V_max_ values were calculated from a Michaelis-Menten plot using Graph Pad Prism 5 software.

## Results and discussion

### Isolation and identification of bacterial isolates

In this study, eighty-one aerobic bacterial morphotypes were obtained from the gastrointestinal (GI) content of a ruminant goat and a non-ruminant indigenous pig (Zovawk). All isolates were identified by 16S rRNA gene sequencing, and all sequences were deposited in the NCBI GenBank and accession numbers were obtained (S1 Table). Out of the 81 isolates, 52 (64.19%) were recovered from the goat GI content and 29 (35.80%) were isolated from the pig GI extract. Among the nutritional media used, actinomycetes isolation agar (AIA) was the most suitable medium, followed by starch casein agar (SCA), Luria Bertani (LB) medium and tryptone soy agar (TSA), which resulted in recoveries of 29, 25, 19 and 8 isolates, respectively (Table 1).

**Table 1:** Frequency of the bacterial isolates obtained from the gastro-intestinal extract of the tested animals using various growth media.

Organisms	Media			
	AIA	SCA	LB	TSA
Goat	19	16	13	4
Pig	10	9	6	4

### Phylogenetic affiliation of the isolates

**Fig 1:**
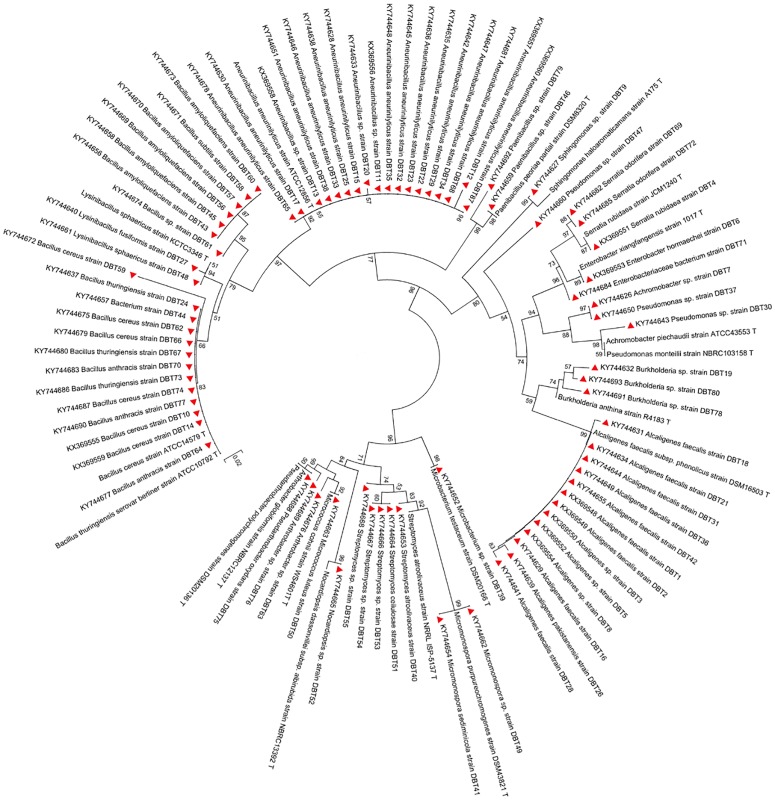
Neighbor-joining phylogenetic tree based on 16S rRNA gene of the bacterial population associated with gastro intestine extract of swine and goat. Numbers at branches indicate bootstrap values of neighbour-joining analysis (>50%) from 1,000 replicates.

All the isolates were identified based on 16S rRNA gene sequencing and was sequenced commercially at Sci-genome labs Pvt. limited, Cochin, India. The desired amplicon size of 700–1200 bp was obtained in all the isolates. The sequences were aligned by BLAST analysis along with the type strains downloaded from EzTaxon-database. The results showed that all the isolates were classified into 12 families belongs to *Bacillaceae* (n = 22; 27.1%) followed by *Paenibacillaceae* (n = 20; 24.6%), *Alcaligenaceae* (n = 14; 17.2%), *Enterobacteriaceae* (n = 5; 6.2%), *Streptomycetaceae* (n = 5; 6.1%), *Micrococcaceae* (n = 4; 4.9%), *Burkholderiaceae* (n = 3; 3.7%), *Pseudomonadaceae* (n = 3; 3.7%), *Micromonosporaceae* (n = 2; 2.4%), *Sphingomonadaceae* (n = 1; 1.2%), *Mycobacteriaceae* (n = 1; 1.2%) *and Nocardiopsaceae* (n = 1; 1.2%). The 16SrRNA gene sequence analysis by BlastN with 97–100% similarity and 25 isolates were divided into gram-negative and 56 isolates were divided into gram positive bacteria. The phylogenetic tree was constructed based on Neighbor-joining method with Kimura 2-parameter model (R = 1.45) according to lowest BIC (4497.89) and highest AIC (2987.72) values using Mega 6.06 **(**Fig 1). Gaps were treated by pair wise deletion and the estimated Transition/Transversion bias (R) is 1.45. The phylogenetic tree revealed that all the gut intestine bacterial strains were separated into three clade (Clade I, Clade II and Clade III). In clade I, all the gram positive bacterial genus *Paenibacillus sp*., *Aneurinibacillus aneurinilyticus*, *Aneurinibacillus sp*., *Bacillus subtilis*, *Bacillus amyloliquefaciens*, *Bacillus sp*., *Lysinibacillus fusiformis*, *Lysinibacillus sphaericus*, *Bacillus cereus*, *Bacillus thuringiensis*, *Bacillus anthracis* and *Bacterium* under bootstrap supported value 77%. Similarly, in clade II, all the gram negative bacterial genus *Alcaligenes*, *Burkholderia*, *Pseudomonas*, *Achromobacter*, *Enterobacter*, *Serratia* and *Sphingomona* are closely clustered with their type strains under high bootstrap value of 80%. Moreover, in clade III contains all the actinobacterial genus *Streptomyces*, *Microbacterium*, *Micromonospora*, *Micrococcus*, *Nocardiopsis*, *Arthrobacter* and *Pseudoarthrobacter* are closely clustered with their type strains retrieved from EzTaxon database under high bootstrap value of 96%. The isolates *Aneurinibacillus aneurinilyticus* strain DBT87 and *Bacillus cereus* strain DBT10 closely clustered with their type strains, *Aneurinibacillus aneurinilyticus* ATCC12856^T^ and *Bacillus cereus* ATCC14579^T^ under bootstrap supported values of 99 and 78%, respectively. Many previous studies have suggested that *Bacteroidetes* and *Firmicutes* are the predominant phyla in the gastrointestianl tracts of warm blooded animals [36, 37]. In our study, *Proteobacteria* was found to be the dominant phylum (n = 69; 85.18%) followed by *Actinobacteria* (n = 12; 14.8%). *Proteobacteria* was also reported as the dominant phylum, followed by *Actinobacteria*, *Spirochaetes* and *Synergistetes*, in the guts of various swine and goat varieties [38, 39].

### Screening for cellulase and xylanase production

Out of 81 isolates, 13 showed promising hydrolysis zones for xylanase, whereas 5 isolates were positive for cellulase production using a Congo red assay (Table 2).

**Table 2:** Production of cellulase and xylanase using Congo red assay by selected bacterial isolates.

Isolate	[A] Size of the colony (cm)	[B] Destained Zone (cm)	[B/A] Hydrolysis capacity
Cellulase			
DBT4 (*S*. *rubidaea*)	1.5	1.2	0.8
DBT87 (*A*. *aneurinilyticus*)	2.0	0.2	0.1
DBT9 (*Sphingomonas* sp.)	1.7	0.7	0.4
DBT15 (*A*. *aneurinilyticus*)	1.9	0.2	0.1
DBT14 (*B*. *cereus*)	1.6	0.4	0.8
Xylanase			
DBT1 (*A*. *faecalis*)	2.5	1.0	0.4
DBT2 (*A*. *faecalis*)	2.0	1.0	0.5
DBT3 (*Alcaligenes* sp.)	2.0	0.5	0.2
DBT10 (*B*. *cereus*)	1.5	1.2	0.8
DBT5 (*Alcaligenes* sp.)	2.0	1.0	0.5
DBT6 (*E*. *hormaechei*)	1.6	0.4	0.2
DBT11 (*Aneurinibacillus* sp.)	1.5	0.3	0.2
DBT8 (*Alcaligenes* sp.)	2.0	1.0	0.5
DBT12 (*A*. *aneurinilyticus*)	2.4	1.2	0.5
DBT13 (*Aneurinibacillus* sp.)	2.9	0.6	0.2
DBT7 (*Achromobacter* sp.)	2.4	1.2	0.5
DBT4 (*S*. *rubidaea*)	1.0	2.0	2.0
DBT87 (*A*. *aneurinilyticus*)	1.0	1.0	1.0

Among the thirteen xylanase-positive bacteria, nine and four isolates were isolated from the goat and swine GI tract extracts, respectively. Similarly, among the cellulase-positive isolates, three and two isolates were from the swine and goat GI extracts, respectively. Among all strains testing positive for cellulase and xylanase production, four (DBT4, DBT87, DBT14 and DBT10) were selected for subsequent studies based on their positive cellulase and xylanase activities. Among them, only two isolates, *Serratia rubidaea* strain DBT4 and *Aneurinibacillus aneurinilyticus* strain DBT87, showed both cellulase and xylanase activity. Anand and Sripathi [40] previously obtained 14 bacterial isolates the from gut intestine of the Indian flying fox (*Pteropus giganteus*), of which eight isolates showed both cellulase and xylanase activity. Anand and Sripathi [40] also reported that a strain of *Serratia liquefaciens* produced both cellulase and xylanase enzymes. Similarly, Gupta et al. [41] reported that strains CDB 8 and CDB 10 showed the highest clear zones around colonies, with diameters of 4.5 and 5.0 cm and hydrolytic values of 9 and 9.8, respectively. The selection of these strains was also based on highest enzyme production in submerged state fermentation, which is discussed in a later section.

### Phylogenetic affiliation of hollocellulolytic bacterial strains

The selected bacterial strains were compared based on the sequence variation in their 16S rRNA gene sequences. The sequence analysis showed that the strains DBT4, DBT87 DBT14 and DBT10 were phylogenetically related to the genera *Serratia*, *Aneurinibacillus* and *Bacillus*, respectively. Based on their sequence similarities with the type strains in NCBI GenBank, the isolates were designated *Serratia rubidaea* DBT4, *Aneurinibacillus aneurinilyticus* DBT87, *Bacillus cereus* DBT14 and *Bacillus cereus* DBT10. To the best of our knowledge, this is the first report of two species, *Pseudoarthrobacter oxydans* strain DBT75 and *Micrococcus luteus* strain DBT50, isolated from gut intestine of Goat.

### Quantification and optimization of enzyme production conditions

Based on their *in vitro* enzymatic assays, four strains (*S*. *rubidaea* DBT4, *A*. *aneurinilyticus* DBT87, *Aneurinibacillus* sp. DBT14 and *Bacillus cereus* DBT10) were selected for further characterization of their enzyme production capabilities using submerged fermentation (SmF) with different substrates.

### Effect of incubation time on cellulase and xylanase production

**Fig 2:**
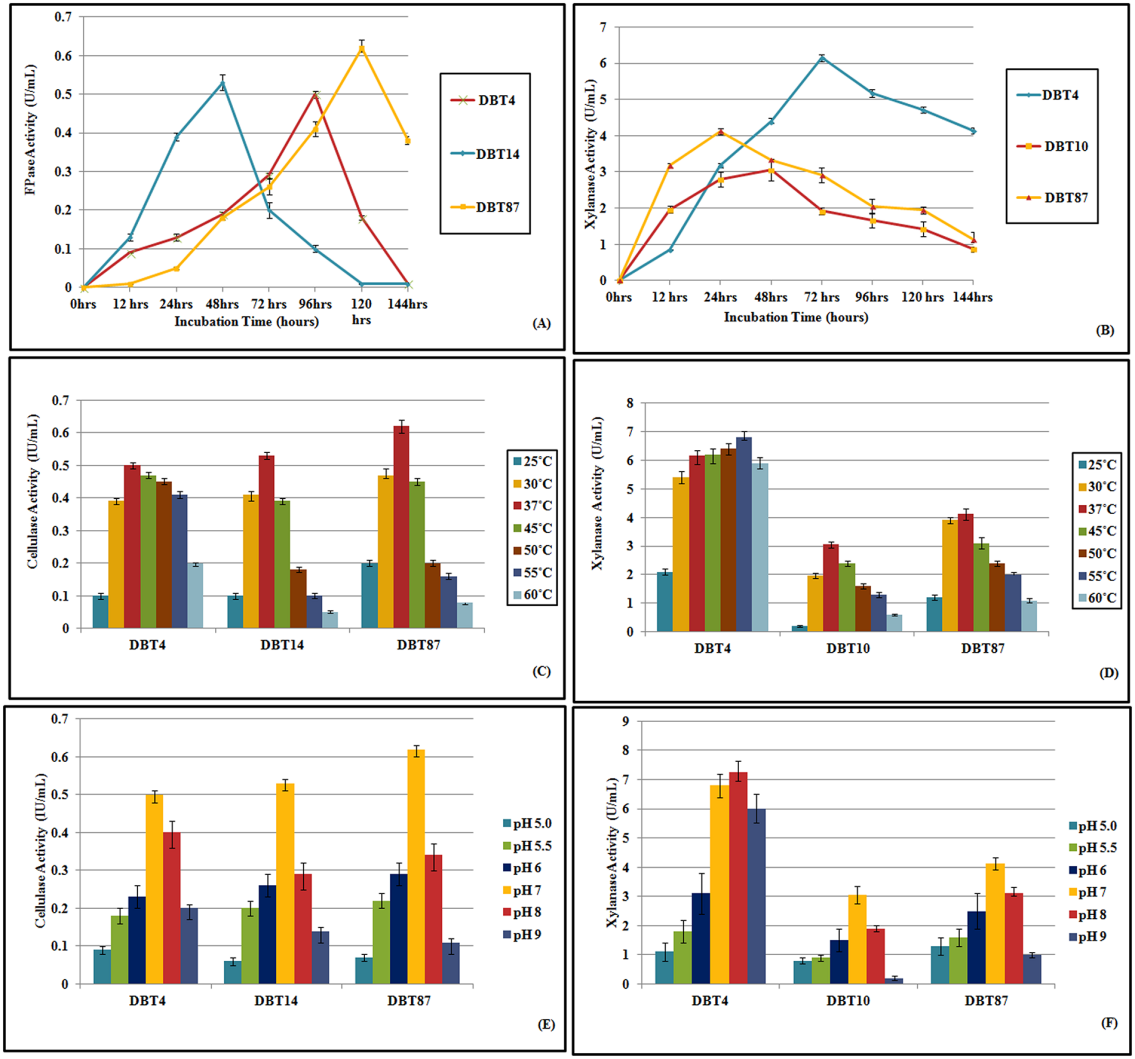
Effect of incubation time, temperature and pH on FPase activity (A, B, C) and on xylanase activity (C, D, F) activity by using three selected bacterial isolates.

The SmF studies, which were carried out for varying incubation times, was performed at ambient temperature and neutral pH to evaluate the growth parameters of the selected bacterial strains and concurrently determine the optimal time required for enzyme productions. Among the cellulase producers, *A*. *aneurinilyticus* DBT87 exhibited the best FPase activity (0.62 U/mL at 120 h), followed by *Aneurinibacillus* sp. DBT14 and *S*. *rubidaea* DBT4, which had FPase activities of 0.53 and 0.5 U/mL at 48 and 96 h, respectively (Fig 2A). This finding was similarly reported by Yang et al. [42], who observed that strain *Bacillus subtilis* BY-2 showed a maximal cellulase activity (CMCase) of 3.56 U/mL within 48 h. In addition, Gupta et al. [41] reported that eight bacterial isolates (CDB1, 2, 3, 6, 7, 8, 9 and 10) showed cellulase production ranging from 0.012 to 0.196 IU/mL for FPC (filter paper cellulase) activity. The xylanase assay revealed that *S*. *rubidaea* DBT4 showed a maximal xylanase activity of 6.15 U/mL at 72 h, followed by *A*. *aneurinilyticus* DBT87 (4.12 U/mL at 96 h) and *Bacillus cereus* DBT10 (3.05 U/mL at 48 h) (Fig 2B). Interestingly, two bacterial strains (*S*. *rubidaea* DBT4 and *A*. *aneurinilyticus* DBT87) showed both cellulase and xylanase activities, with the xylanase produced earlier (at 72 and 48 h, respectively) followed by cellulase (at 120 and 96 h, respectively). This suggested that they might be ideal microbes for biomass utilization, as the xylanase expression will initially allow result in the hydrolysis of the hemicelluloses linkages, possibly helping to free up the cellulosic compounds that could then be acted upon by the cellulases.

### Effect of temperature on cellulase and xylanase production

The temperature profiles were established at the optimal incubation times for the three cellulase producing strains and at neutral pH. The results showed that the optimal temperature for enzyme production for the three strains was at 37°C. Interestingly, *S*. *rubidaea* DBT4 had an FPase activity of 0.41 U/mL at 55°C (Fig 2C). Similar results were observed for the two xylanase producing strains (*A*. *aneurinilyticus* DBT87 and *Bacillus cereus* DBT10). *S*. *rubidaea* DBT4 showed an enhanced xylanase activity of 6.8 U/mL at 55°C compared to 37°C but decreased at 60°C (Fig 2D). This finding was consistent with the findings of Seo et al. [43] who observed that *Bacillus licheniformis* strain JK7 showed maximal xylanase activity (0.44 U/mL) at 55°C. The optimum temperatures for xylanases from various *Bacillus* sp. have also been previously reported [19, 44–47]. Moreover, Choi et al. [48] reported that *Bacillus licheniformis* strain KCTC1918 had xylanase activity at 47°C. Furthermore, *Bacillus* strain DUSELR13 also demonstrated maximal endoglucanase activity at 75°C as described by Rastogi et al. [49]. This result indicated that *S*. *rubidaea* DBT4 might be a thermo-tolerant bacterial strain, with both xylanase and cellulase producing capabilities. There are very few reports in the Uniport database of xylanases similar to β-xylosidase (http://www.uniprot.org/uniprot/A0A126VGJ6), endo-1, 4-beta-xylanase Z (http://www.uniprot.org/uniprot/A0A126VIN5) or for the gene encoding the cellulase 6-phospho-beta-glucosidase (http://www.uniprot.org/uniprot/A0A126VIF7) in strain S. *rubidaea*. Thermophilic cellulose degrading enzymes have great potential for the biofuel, leather, textile, food and agriculture industries, since high temperatures are often required in these processes [50, 49].

### Effect of pH on cellulase and xylanase production

Finally, using the optimized incubation time of growth and temperature, the initial pH was varied for SmF conditions. The best pH for the growth of the two cellulase and two xylanase producers was pH 7, whereas for *S*. *rubidaea* DBT4, the xylanase production was higher at pH 8 (7.25 U/mL) and an FPase activity of 0.4 U/mL (Fig 2E and 2F) was obtained at this alkaline condition. This result indicates that the strain *S*. *rubidaea* DBT4 was able to produce enzymes with substantial activity at the alkaline pH of 8. This findings was similarly reported by Seo et al. [43], where a *Bacillus licheniformis* isolate from the rumen of a Korean goat exhibited a maximal xylanase activity of 1.08 U/mL at pH 5.0. Immanuel et al. [51] reported that cellulolytic endoglucanase enzymes from *Cellulomonas*, *Bacillus*, and *Micrococcus* sp., which were isolated from estuarine coir netting effluents, hydrolyzed substrate in a pH range of 4.0 to 9.0, with maximal activity at pH 7.0. *Bacillus* sp. MVS3 showed a maximal FPase production at pH 7.0 [52].

### Biomass compositional analysis

Given the pulpy nature of the grass variety used in this study, the use of this grass as a biomass was done in such a way that the pulpy nature could be retained. In order to generate a homogenized pulpy biomass, an alkali treatment was performed and the effect of this mild pretreatment was confirmed using compositional analysis. The results showed an enhancement in the cellulose content, while the hemicelluloses and lignin contents decreased from the raw biomass to the alkali pretreated biomass. This increase in cellulosic content by the alkali treatment (from 48.74 to 55.85%) could be due to the decrease in the hemicellulosic compounds (17.74 to 12.22%) upon treatment (Table 3). Because of the decreased lignin content and the high cellulosic contents, this biomass in its pulpy form would be an ideal, non-edible biomass feedstock.

**Table 3:** Compositional analysis of BPS-G104 (raw *T*. *latifolia*) and BPS-G104a (alkaline pretreated pulpy *T*. *latifolia*).

Sample name	Cellulose (%)*	Hemicellulose (%)*	Lignin (%)*	Moisture content (%)*	Extractives (%)*	Ash (%)*	Reference
BPS-G-104	48.74 ± 0.65	17.74 ± 0.71	8.7 ± 0.62	8.12 ± 0.12	16.26 ± 0.34	0.44 ± 0.01	Leo et al., 2016
BPS-G-104a	55.85 ± 0.13	13.22 ± 0.32	6.92 ± 0.81	5.47 ± 0.23	12.3 ± 0.34	0.25 ± 0.53	This study

*Each value represents the value of three replicates, Mean (±) SD

### Biomass utilization potential

**Fig 3:**
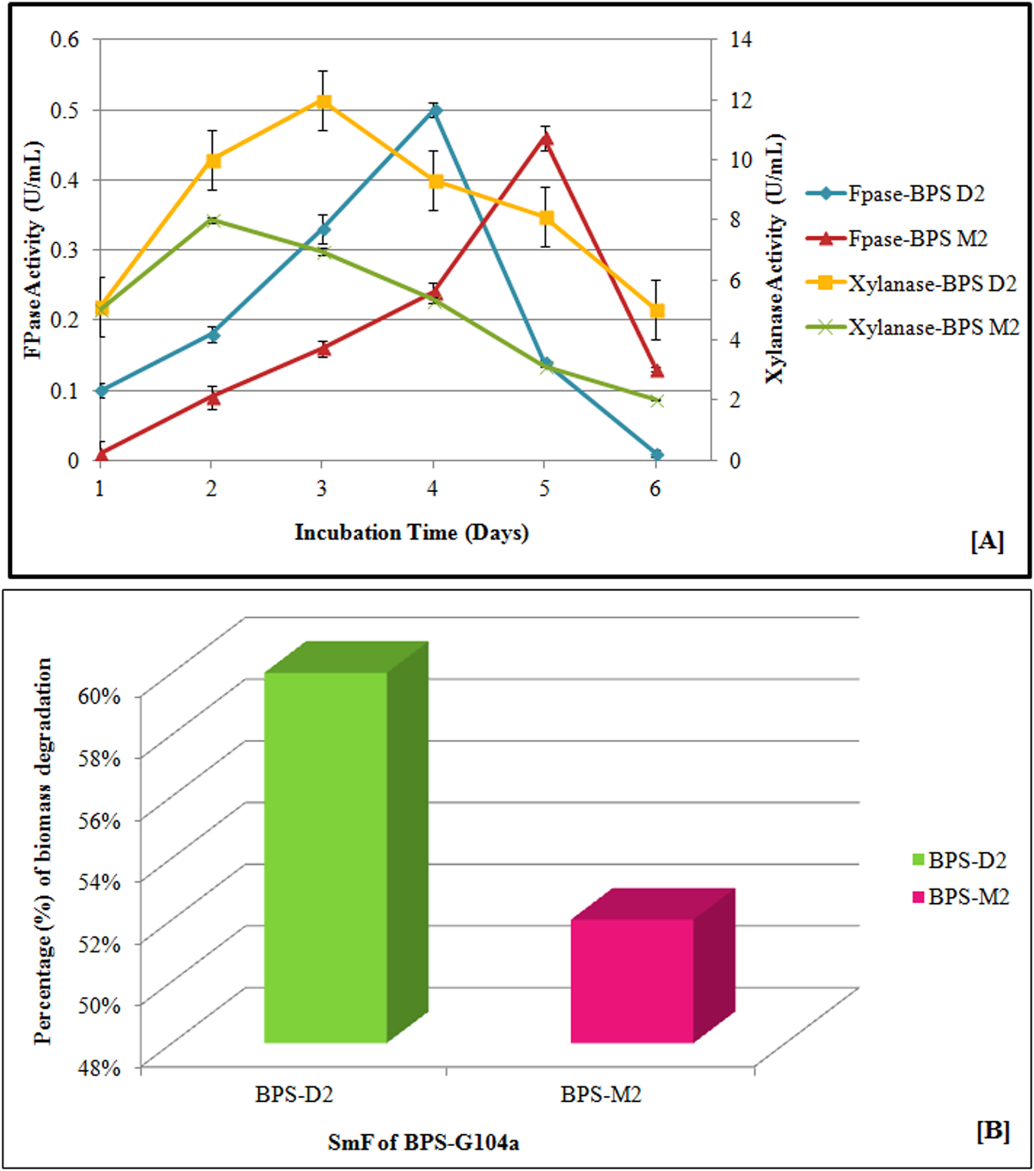
(A) Effect of incubation time on FPase and xylanase activity of treatment BPS-D2 and BPS-M2 under submerged fermentation (SmF) at optimized temperature and pH. (B) Percentage of biomass degradation by using different treatments.

*A*. *aneurinilyticus* DBT87 and *S*. *rubidaea* DBT4, which both exhibited cellulase and xylanase activity, were selected to evaluate their biomass utilization potential using a perennial grass, *T*. *latifolia* (BPS-G104a). From the experimental design, treatment BPS-D2 was the most efficient, with maximal xylanase (11.98 U/mL) and cellulase (0.5 U/mL) production on 3^rd^ and 4^th^ days, respectively. Consistent xylanase production at 55°C and pH 8 was observed, with a range from 8.13 to 11.98 U/mL from the 2^nd^ to the 5^th^ day of incubation. This suggests that *S*. *rubidaea* DBT4 is capable of efficiently utilizing the hemicellulose content of BPS-G104a and could be the reason for the enhanced xylanase production. Similarly, a slight increase in FPase activity (0.4 to 0.5 U/mL) was also observed in BPS-D2, further showing the bioconversion potential of the strain. Cellulase production was more prominent in the treatment M2, with an optimal FPase production of 0.70 U/mL observed on the 5^th^ day, even though the xylanase production was not as prominent as BPS-D2 (Fig 3A). With the ability to steadily produce xylanase and the concurrent production of cellulase at thermophilic and alkaline conditions, *S*. *rubidaea* DBT4 will be an exploitable source for the bioconversion of pulpy biomasses such as BPS-G104 with less lignin content. In all the treatments, a 40 to 60% loss in dry weight was observed, with a maximum of 60% using BPS-D2 followed by BPS-M2 compared to the alkali pretreated biomass that served as the control (Fig 3B).

### FTIR analysis

**Fig 4:**
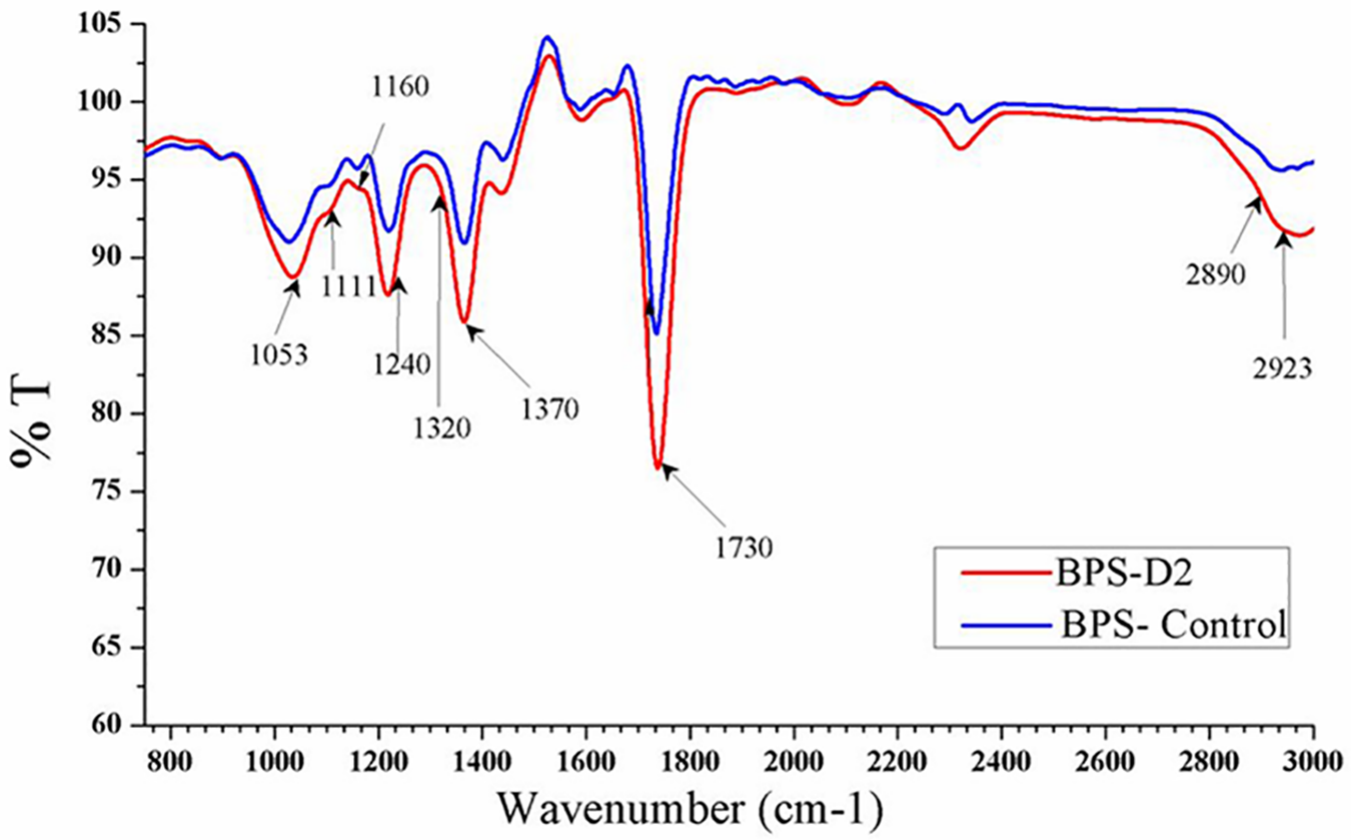
FTIR analysis of the most potential treatment (BPS-D2) as compare to the pre-treated control (BPS-Control).

To observe any structural alterations that occurred during the two most efficient treatments (BPS-D2 and BPS-M2) with respect to the alkali pretreated biomass, samples were analyzed using FTIR spectral readings ranging from 700 to 3000 cm^−1^. Significant variation in the peaks corresponding to 1053 cm^-1^ were observed for both BPS-D2 and BPS-M2 treatments, indicating C-O stretching for crystalline cellulose or hemicellulose. At 1370 cm^-1^, which is the characteristic wavelength for crystalline cellulose symmetric C-H deformation, the BPS-D2 treatment showed a more prominent reduction in the peak compared with the BPS-M2 treatment. A similar variation was observed at 1240 cm^-1^, indicative of the C-O stretching for xylan. Characteristic peak assignments for hemicellulose (xylan) at 1735/1730 cm^−1^ (unconjugated C = O stretching) were observed, with considerable variation in peak reduction in the BPS-D2 treatment (63%) compared to BPS-M2 (76%) (Fig 4). This suggested that the xylan has been utilized efficiently in the BPS-D2 treatment, which is line with our biomass utilization assay results. The C-H stretching of lignocellulose components detected at 2890/2923 cm^-1^ also varied between the BPS-D2 and BPS-M2 treatments, with BPS-D2 being more effective. Thus, the FTIR analysis, which was performed to determine the impact of the BPS-D2 and BPS-M2 treatments on the buildup of holocellulosic components, revealed that the BPS-D2 treatment was more successful in reducing the biomass macromolecules. In the current study, slight variations in FTIR readings were observed at 1053, 1240, 1735/1730 cm^-1^, similar to what was observed in other studies [1, 53]. An understanding of the chemical components in the biomass is tremendously important for determining their viability as a potent substrates for biofuel production.

### Scanning electron microscopy (SEM) analysis

**Fig 5:**
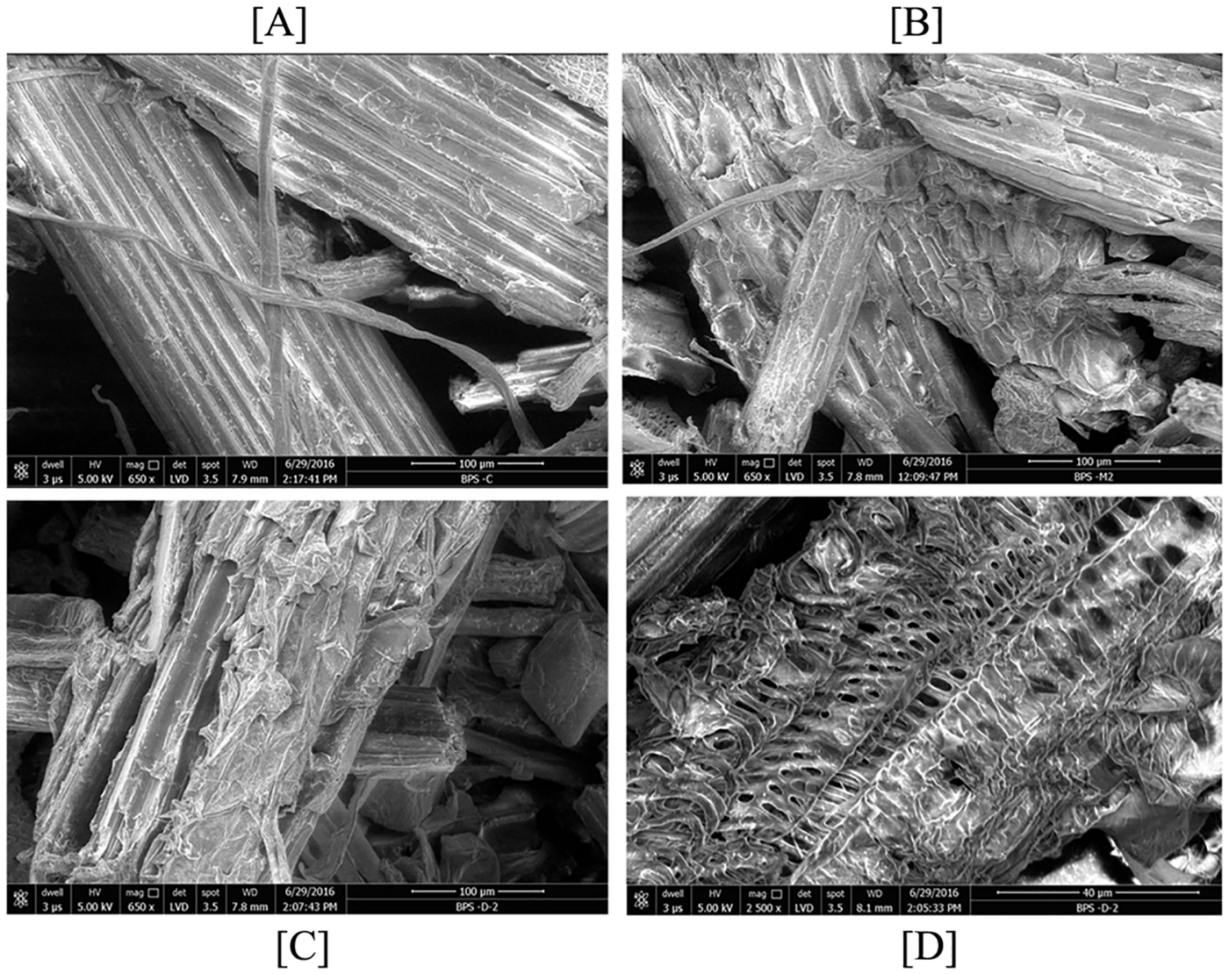
Surface destruction images of *T*. *latifolia* (BPS-G104) using SEM. (A) pre-treated biomass as control (BPS-C); (B) biomass treated with *Aneurinibacillus aneurinilyticus* strain DBT87 (BPS-M2); (C) and (D) biomass treated with *Serratia rubidaea* strain DBT4 (BPS-D2).

The two best treatments were chosen for a topology analysis using SEM after 6 days of SmF, along with an alkali pre-treated biomass sample as a control. Both treatments showed disintegration and breakdown of the fibrous holocellulosic components as well as gaps and sutures in the biomass layer (Fig 5). The SEM analysis confirmed the extent of enzyme action on the biomass and its effect on biomass morphology.

### Kinetic study of the BPS-DBT4 xylanase enzyme

**Fig 6:**
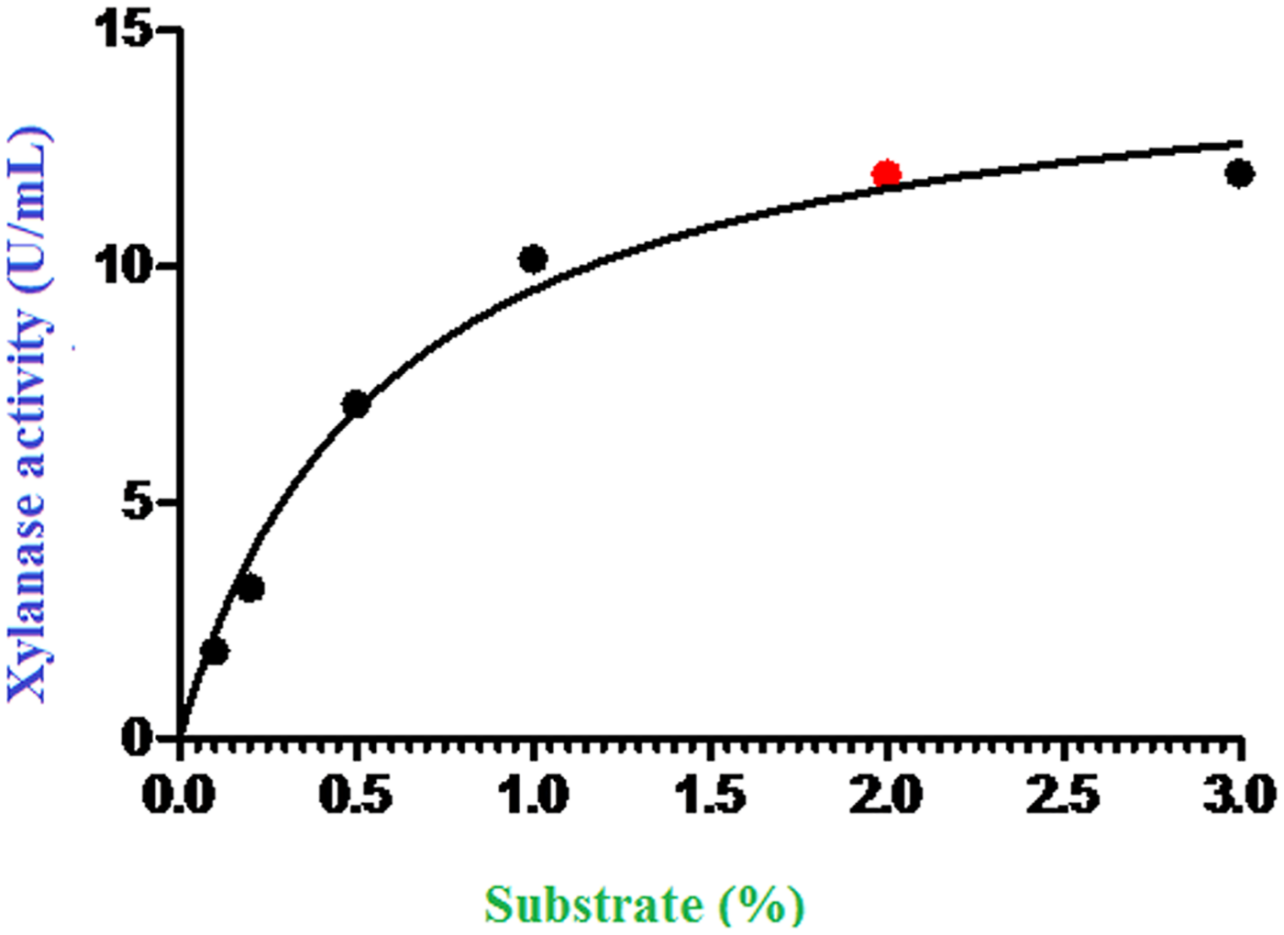
Kinetic analysis of xylanase obtained from submerged fermentation of treatment BPS-D2 and its hydrolyzing effect on varying concentrations of beechwood xylan.

The kinetic capability of the optimized crude xylanase obtained from the SmF treatment of BPS-G104 by *S*. *rubidaea* DBT4 was verified under varying substrate (beechwood xylan) concentrations (0.1, 0.2, 0.5, 1, 2 and 3%) in a xylanase DNS assay. The kinetic study of the *S*. *rubidaea* DBT4 xylanase revealed that the V_max_ was as high as 12.56 U/mL and a stable K_m_ for the enzyme (0.58) was observed (Fig 6).

## Conclusions

This study sought to determine the potential of gut or ruminal bacteria for holocellulose degrading enzymes. The identified enzymes could have the additional benefits of surviving in harsher conditions (alkaline, thermophillic) and being substrate specific (holocellulosic biomass). The detection of these microbes has significant implications for their use in the degradation of lignocelluloses biomass and typifies potential targets for novel enzymes with industrial applications.

## Supporting information

S1 Table Identification of bacterial strains isolated from animal gut based on 16S rRNA gene sequences similarity compared with closest type strains. (DOC)
