# Supplementary material for: Evaluation of gastrointestinal bacterial population for the production of holocellulose enzymes for biomass deconstruction
Source: PLoS One. 2017 Oct 12;12(10):e0186355. doi: 10.1371/journal.pone.0186355 (PMC5638507; doi:10.1371/journal.pone.0186355)
Supplement: S1 Table — (DOC) [file pone.0186355.s001.doc]

**Table S1. Identification of bacterial strains isolated from animal gut based on 16S rRNA gene sequences similarity compared with closest type strains**

| **Sl. No** | **Strain Name** | **Isolates Name** | **Accession Number** | **Similarity Strain** | **Identity** |
| --- | --- | --- | --- | --- | --- |
| 1 | DBT1 | *Alcaligenes faecalis* | KX369548 | *Alcaligenes faecalis* subsp. *phenolicus* strain DSM16503 | 100% |
| 2 | DBT2 | *Alcaligenes faecalis* | KX369549 | *Alcaligenes faecalis* subsp. *phenolicus* strain DSM16503 | 99% |
| 3 | DBT3 | *Alcaligenes* sp. | KX369550 | *Alcaligenes faecalis* subsp. *phenolicus* strain DSM16503 | 98% |
| 4 | DBT4 | *Serratia rubidaea* | KX369551 | *Serratia rubidaea* strain JCM1240 | 99% |
| 5 | DBT5 | *Alcaligenes* sp. | KX369552 | *Alcaligenes faecalis* subsp. *phenolicus* strain DSM16503 | 99% |
| 6 | DBT6 | *Enterobacter hormaechei* | KX369553 | *Enterobacter xiangfangensis* strain 1017 | 97% |
| 7 | DBT7 | *Achromobacter* sp. | KY744626 | *Achromobacter piechaudii* strain ATCC43553 | 99% |
| 8 | DBT8 | *Alcaligenes* sp. | KX369554 | *Alcaligenes faecalis* subsp. *phenolicus* strain DSM16503 | 99% |
| 9 | DBT9 | *Sphingomonas* sp. | KY744627 | *Sphingomonas haloaromaticamans* strain A175 | 99% |
| 10 | DBT10 | *Bacillus cereus* | KX369555 | *Bacillus cereus* strain ATCC14579 | 99% |
| 11 | DBT11 | *Aneurinibacillus*  sp. | KX369556 | *Aneurinibacillus aneurinilyticus* strain ATCC12856 | 99% |
| 12 | DBT12 | *Aneurinibacillus aneurinilyticus* | KX369557 | *Aneurinibacillus aneurinilyticus* strain ATCC12856 | 99% |
| 13 | DBT13 | *Aneurinibacillus* sp. | KX369558 | *Aneurinibacillus aneurinilyticus* strain ATCC12856 | 99% |
| 14 | DBT14 | *Bacillus cereus* | KX369559 | *Bacillus cereus* strain ATCC14579 | 100% |
| 15 | DBT15 | *Aneurinibacillus aneurinilyticus* | KY744628 | *Aneurinibacillus aneurinilyticus* strain ATCC12856 | 99% |
| 16 | DBT16 | *Alcaligenes faecalis* | KY744629 | *Alcaligenes faecalis* subsp. *phenolicus* strain DSM16503 | 99% |
| 17 | DBT17 | *Aneurinibacillus aneurinilyticus* | KY744630 | *Aneurinibacillus aneurinilyticus* strain ATCC12856 | 99% |
| 18 | DBT18 | *Alcaligenes faecalis* | KY744631 | *Alcaligenes faecalis* subsp. *phenolicus* strain DSM16503 | 99% |
| 19 | DBT19 | *Burkholderia* sp. | KY744632 | *Burkholderia anthina* strain R4183 | 97% |
| 20 | DBT20 | *Aneurinibacillus aneurinilyticus* | KY744633 | *Aneurinibacillus aneurinilyticus* strain ATCC12856 | 99% |
| 21 | DBT21 | *Alcaligenes faecalis* | KY744634 | *Alcaligenes faecalis* subsp. *phenolicus* strain DSM16503 | 99% |
| 22 | DBT22 | *Aneurinibacillus aneurinilyticus* | KY744635 | *Aneurinibacillus aneurinilyticus* strain ATCC12856 | 99% |
| 23 | DBT23 | *Aneurinibacillus aneurinilyticus* | KY744636 | *Aneurinibacillus aneurinilyticus* strain ATCC12856 | 99% |
| 24 | DBT24 | *Bacillus thuringiensis* | KY744637 | *Bacillus thuringiensis serovar* Berliner strain ATCC10792 | 100% |
| 25 | DBT25 | *Aneurinibacillus aneurinilyticus* | KY744638 | *Aneurinibacillus aneurinilyticus* strain ATCC12856 | 99% |
| 26 | DBT26 | *Alcaligenes pakistanensis* | KY744639 | *Alcaligenes faecalis* subsp. *phenolicus* strain DSM16503 | 99% |
| 27 | DBT27 | *Lysinibacillus fusiformis* | KY744640 | *Lysinibacillus sphaericus* strain KCTC3346 | 99% |
| 28 | DBT28 | *Alcaligenes faecalis* | KY744641 | *Alcaligenes faecalis* subsp. *phenolicus* strain DSM16503 | 98% |
| 29 | DBT29 | *Aneurinibacillus aneurinilyticus* | KY744642 | *Aneurinibacillus aneurinilyticus* strain ATCC12856 | 99% |
| 30 | DBT30 | *Pseudomonas* sp. | KY744643 | *Pseudomonas monteilii* strain NBRC 103158 | 97% |
| 31 | DBT31 | *Alcaligenes faecalis* | KY744644 | *Alcaligenes faecalis* subsp. *phenolicus* strain DSM16503 | 99% |
| 32 | DBT32 | *Aneurinibacillus aneurinilyticus* | KY744645 | *Aneurinibacillus aneurinilyticus* strain ATCC12856 | 99% |
| 33 | DBT33 | *Aneurinibacillus aneurinilyticus* | KY744646 | *Aneurinibacillus aneurinilyticus* strain ATCC12856 | 99% |
| 34 | DBT34 | *Aneurinibacillus aneurinilyticus* | KY744647 | *Aneurinibacillus aneurinilyticus* strain ATCC12856 | 99% |
| 35 | DBT35 | *Aneurinibacillus aneurinilyticus* | KY744648 | *Aneurinibacillus aneurinilyticus* strain ATCC12856 | 99% |
| 36 | DBT36 | *Alcaligenes faecalis* | KY744649 | *Alcaligenes faecalis* subsp. *phenolicus* strain DSM16503 | 99% |
| 37 | DBT37 | *Pseudomonas* sp. | KY744650 | *Pseudomonas monteilii* strain NBRC 103158 | 97% |
| 38 | DBT38 | *Aneurinibacillus aneurinilyticus* | KY744651 | *Aneurinibacillus aneurinilyticus* strain ATCC12856 | 99% |
| 39 | DBT39 | *Microbacterium* sp. | KY744652 | *Microbacterium testaceum* strain DSM20166 | 98% |
| 40 | DBT40 | *Streptomyces atroolivaceus* | KY744653 | *Streptomyces atroolivaceus* strain NRRL ISP-5137 | 99% |
| 41 | DBT41 | *Micromonospora sediminicola* | KY744654 | *Micromonospora purpureochromogenes* strain DSM43821 | 97% |
| 42 | DBT42 | *Alcaligenes faecalis* | KY744655 | *Alcaligenes faecalis* subsp. *phenolicus* strain DSM16503 | 99% |
| 43 | DBT43 | *Bacillus amyloliquefaciens* | KY744656 | *Bacillus cereus* strain ATCC14579 | 99% |
| 44 | DBT44 | *Bacterium* | KY744657 | *Bacillus cereus* strain ATCC14579 | 99% |
| 45 | DBT45 | *Bacillus amyloliquefaciens* | KY744658 | *Bacillus cereus* strain ATCC14579 | 99% |
| 46 | DBT46 | *Paenibacillus* sp. | KY744659 | *Paenibacillus peoriae* strain DSM8320 | 98% |
| 47 | DBT47 | *Pseudomonas* sp. | KY744660 | *Pseudomonas monteilii* strain NBRC 103158 | 99% |
| 48 | DBT48 | *Lysinibacillus sphaericus* | KY744661 | *Lysinibacillus sphaericus* strain KCTC3346 | 98% |
| 49 | DBT49 | *Micromonospora* sp. | KY744662 | *Micromonospora purpureochromogenes* strain DSM43821 | 99% |
| 50 | DBT50 | *Micrococcus luteus* | KY744663 | *Micrococcus cohnii* strain WS4601 | 99% |
| 51 | DBT51 | *Streptomyces cellulosae* | KY744664 | *Streptomyces atroolivaceus* strain NRRL ISP-5137 | 99% |
| 52 | DBT52 | *Nocardiopsis* sp. | KY744665 | *Nocardiopsis dassonvillei* subsp. *albirubida* strain NBRC13392 | 99% |
| 53 | DBT53 | *Streptomyces* sp. | KY744666 | *Streptomyces atroolivaceus* strain NRRL ISP-5137 | 99% |
| 54 | DBT54 | *Streptomyces* sp. | KY744667 | *Streptomyces atroolivaceus* strain NRRL ISP-5137 | 99% |
| 55 | DBT55 | *Streptomyces* sp. | KY744668 | *Streptomyces atroolivaceus* strain NRRL ISP-5137 | 99% |
| 56 | DBT56 | *Bacillus amyloliquefaciens* | KY744669 | *Bacillus cereus* strain ATCC14579 | 97% |
| 57 | DBT57 | *Bacillus amyloliquefaciens* | KY744670 | *Bacillus cereus* strain ATCC14579 | 97% |
| 58 | DBT58 | *Bacillus subtilis* | KY744671 | *Bacillus cereus* strain ATCC14579 | 98% |
| 59 | DBT59 | *Bacillus cereus* | KY744672 | *Bacillus cereus* strain ATCC14579 | 98% |
| 60 | DBT60 | *Bacillus amyloliquefaciens* | KY744673 | *Bacillus cereus* strain ATCC14579 | 97% |
| 61 | DBT61 | *Bacillus amyloliquefaciens* | KY744674 | *Bacillus cereus* strain ATCC14579 | 97% |
| 62 | DBT62 | *Bacillus cereus* | KY744675 | *Bacillus cereus* strain ATCC14579 | 100% |
| 63 | DBT63 | *Arthrobacter* sp. | KY744676 | *Arthrobacter globiformis* strain NBRC12137 | 99% |
| 64 | DBT64 | *Bacillus anthracis* | KY744677 | *Bacillus cereus* strain ATCC14579 | 97% |
| 65 | DBT65 | *Aneurinibacillus aneurinilyticus* | KY744678 | *Aneurinibacillus aneurinilyticus* strain ATCC12856 | 99% |
| 66 | DBT66 | *Bacillus cereus* | KY744679 | *Bacillus cereus* strain ATCC14579 | 100% |
| 67 | DBT67 | *Bacillus thuringiensis* | KY744680 | *Bacillus thuringiensis* serovar *Berliner* strain ATCC10792 | 99% |
| 68 | DBT68 | *Aneurinibacillus aneurinilyticus* | KY744681 | *Aneurinibacillus aneurinilyticus* strain ATCC12856 | 99% |
| 69 | DBT69 | *Serratia odorifera* | KY744682 | *Serratia rubidaea* strain JCM1240 | 97% |
| 70 | DBT70 | *Bacillus anthracis* | KY744683 | *Bacillus cereus* strain ATCC14579 | 98% |
| 71 | DBT71 | *Enterobacteriaceae bacterium* | KY744684 | *Enterobacter xiangfangensis* strain 1017 | 99% |
| 72 | DBT72 | *Serratia odorifera* | KY744685 | *Serratia rubidaea* strain JCM1240 | 98% |
| 73 | DBT73 | *Bacillus thuringiensis* | KY744686 | *Bacillus thuringiensis serovar* Berliner strain ATCC10792 | 99% |
| 74 | DBT74 | *Bacillus cereus* | KY744687 | *Bacillus cereus* strain ATCC14579 | 99% |
| 75 | DBT75 | *Pseudarthrobacter oxydans* | KY744688 | *Pseudarthrobacter polychromogenes* strain DSM20136 | 100% |
| 76 | DBT76 | *Arthrobacter* sp. | KY744689 | *Arthrobacter globiformis* strain NBRC12137 | 99% |
| 77 | DBT77 | *Bacillus anthracis* | KY744690 | *Bacillus cereus* strain ATCC14579 | 97% |
| 78 | DBT78 | *Burkholderia* sp. | KY744691 | *Burkholderia anthina* strain R4183 | 99% |
| 79 | DBT79 | *Paenibacillus* sp. | KY744692 | *Paenibacillus peoriae* strain DSM8320 | 99% |
| 80 | DBT80 | *Burkholderia* sp. | KY744693 | *Burkholderia anthina* strain R4183 | 99% |
| 81 | DBT87 | *Aneurinibacillus aneurinilyticus* | KX369560 | *Aneurinibacillus aneurinilyticus* strain ATCC12856 | 99% |
